# Mesenchymal stromal/stem cells and their exosomes for restoration of spermatogenesis in non-obstructive azoospermia: a systemic review

**DOI:** 10.1186/s13287-021-02295-9

**Published:** 2021-04-06

**Authors:** Rano Zhankina, Neda Baghban, Manarbek Askarov, Dana Saipiyeva, Almaz Ibragimov, Bakhyt Kadirova, Arezoo Khoradmehr, Iraj Nabipour, Reza Shirazi, Ulanbek Zhanbyrbekuly, Amin Tamadon

**Affiliations:** 1 Department of Urology and Andrology, Astana Medical University, Nur-Sultan, Kazakhstan 010000; 2 The Persian Gulf Marine Biotechnology Research Center, The Persian Gulf Biomedical Sciences Research Institute, Bushehr University of Medical Sciences, Bushehr, 7514633196 Iran; 3 Department of Anatomy, School of Medical Sciences, Medicine, UNSW Sydney, PO Box 2052, Sydney, Australia

**Keywords:** Non-obstructive azoospermia, Infertility, Mesenchymal stromal/stem cell, In vitro study, Animal model, Clinical trial

## Abstract

Stem cells have been introduced as new promising therapeutic agents in treatment of degenerative diseases because of having high differentiation potential while maintaining the ability to self-replicate and retaining features of their source cells. Among different type of cell therapies, mesenchymal stromal/stem cell (MSC) therapy is being increasingly developed as a new way to treat structural defects that need to be repaired and regenerated. Non-obstructive azoospermia (NOA) is a reproductive disease in men that causes infertility in 10% of infertile men. Based on in vitro studies, MSCs from different tissue sources have been differentiated into germ cells or gamete progenitor cells by simple methods in both male and female. On the other hand, the therapeutic effects of MSCs have been evaluated for the treatment of NOA animal models created by chemical or surgical compounds. The results of these studies confirmed successful allotransplantation or xenotransplantation of MSCs in the seminiferous tubules. As well, it has been reported that exosomes secreted by MSCs are able to induce the process of spermatogenesis in the testes of infertile animal models. Despite numerous advances in the treatment of reproductive diseases in men and women with the help of MSCs or their exosomes, no clinical trial has been terminated on the treatment of NOA. This systematic review attempts to investigate the possibility of MSC therapy for NOA in men.

## Introduction

In the current decade, the emerging field of stem cell therapy has quickly become a new era of regenerative medicine. The diverse potential of stem cells is a focus of research of many scientists in molecular biology, genetic engineering, and even general medicine for developing new approaches in the treatment of a number of diseases, which have always been a challenge for clinicians [1].

Stem cells are cells that are self-sustaining throughout the life of an organism and capable of differentiating into cells of various types. There are several types of stem cells found in human tissues. Among them, mesenchymal stromal/stem cells (MSCs) derived from various tissues including the bone marrow and adipose tissue have been considered to be the most promising material in terms of their application for cell therapy. The MSCs are popular among scientists and clinicians due to their multilinear differentiation potential, low immunogenicity and active participation in tissue repair, and regeneration after migration to damaged sites. In general, MSCs have advantages over other types of stem cells for clinical use in cell-based therapies. These advantages include availability, easy to isolate and expand, multilineal differentiation, immunosuppressive, and both of the autograft and allograft are possible, free from ethical issues, and limited replicative lifespan [2].

According to World Health Organization (WHO) criteria, the marriage is considered infertile, if no pregnancy occurs within 12 months of unprotected sex [3]. This pathology is an important medico-social issue with up to one in six of married couples failing to conceive naturally [4]. Among them, 20–30% of infertility is related solely to men [5]. Infertility cases are linked to the diminished quantity or quality of ejaculate, which may be due to impaired spermatogenesis, slow maturation of spermatozoa in the epididymis, or incomplete patency of the vas deferens. The main causes of male infertility are genetic disorders, urogenital infections, hypogonadism, cryptorchidism, varicocele, ejaculatory disorders, general and systemic diseases, and immunological factors [6]. Despite its multifactorial nature, male infertility has not been fully understood and about half of cases are considered idiopathic or unexplained [7]. Investigation of male fertility usually starts with history, physical examination, and spermogram.

Azoospermia is classified as obstructive and non-obstructive (NOA). In most patients with NOA, it is possible to distinguish clinically by diagnostic workup including history, hormone levels, and physical examination. These indicators allow to confidently determine the type of azoospermia. This is important, since obstructive azoospermia is more favorable due to preservation spermatogenesis. However, the NOA accounts for about 10% of infertility cases and manifests as the absence of spermatozoa in ejaculates due to spermatogenic deficiency. In the overwhelming majority of cases, azoospermia is associated with a number of irreversible disorders of the testicles, which lead to inhibition of spermatogenesis. Such disorders are most often linked to endocrine, genetic, and inflammatory diseases [8]. In addition, NOA can be idiopathic [9]. Palpation and measurement usually reveal small and flabby testicles typical for non-obstructive azoospermia. In all patients with azoospermia, the levels of follicle stimulating hormone (FSH), luteinizing hormone (LH), prolactin, total testosterone, estradiol, and inhibin B should be measured [10]. In most patients with NOA, FSH is increased (> 7.6 IU/mL) and LH is elevated or close to normal [11]. Hypogonadism is defined by low total testosterone levels (< 300 ng/dL) and occurs in the majority of patients with NOA, usually reflecting Leydig cell deficiency [12]. Obesity can be associated with low total testosterone levels, thereby serum estradiol levels increase due to elevated aromatization of androgens in peripheral tissues [13]. Low testosterone in obese patients may also reflect adaptation to altered sex hormone-binding globulin (SHBG) rather than true testosterone deficiency [14].

Proper counseling and management of patients with NOA presents a challenge for andrologists, urologists, and reproductive medicine specialists. Despite this, advances in molecular biology, hormone replacement therapy, and microsurgical sperm retrieval, together with modern techniques of in vitro fertilization (IVF), give hope for natural paternity. By the way application of MSC for treatment of NOA needs more clarification which this systemic review attempts to do it.

## Methods

### Focused question

This systematic review was done to answer this question: “Could MSCs be applied in treatment of NOA in human?”

### Search and study selection

Key words and subject terms included (“MSC” AND “azoospermia”) OR (“MSC” AND “azoospermia” AND “therapy”) OR (“MSC” AND “germ cell”) OR (“MSC” AND “infertiltiy”) OR (“MSC” AND “reproductive”). The search strategy was applied to Google Scholar and ClinicalTrials.gov, being focused on the in vitro or in vivo studies and clinical trials, respectively. English language publications were considered. The reviews, abstracts without full manuscripts, the manuscripts about non-male reproductive system MSCs therapy, and stem cell sources other than MSCs were excluded. Data were collected from the full text of the articles as follows: (i) the source of MSCs, (ii) type of the study (in vitro, in vivo, or clinical trials), and (iii) the obtained results.

## Treatment of NOA

The NOA has been considered to be a condition not responding to drug therapy [15]. Patients with NOA are unable to have children of their own and have options of either adoption or using donated sperm [16]. Despite the marked changes in spermatogenesis, these patients still have a chance to conceive. In such situations, the preservation of spermatogenesis may be focal in testicular tissues [17]. Due to irreversible nature of spermatogenesis damage in patients with NOA, testicular biopsy and assisted reproductive technologies are the only ways to obtain biological off-springs [18]. For men with NOA, testicular sperm extraction (TESE) with intracytoplasmic sperm injection (ICSI) remains the only choice to conceive [19]. However, TESE-ICSI has limited success in patients with NOA, as during the first TESE cycle, sperm is found about 50% of cases [20], and the subsequent probability of egg fertilization with ICSI is about 50% [21]. As a result, the successful fertilization probability with this technique is about 13.4% [22]. Therefore, considering this low success rate and due to their unlimited source and high differentiation potential, MSCs have been considered as a potential new therapeutic agent for the treatment of infertility.

## MSC-therapy of NOA

The MSCs were first described by Alexander Friedenstein (1924–1998) [23]. He experimentally proved the existence of stromal stem cells in the bone marrow and in lymphoid organs [24]. This discovery confirmed that the bone marrow contains a distinct population of stem cells capable of forming clones of cells of connective and hematopoietic lines [24]. Approximately 30% of the bone marrow aspirate isolated by Friedenstein consisted of MSCs [24]. These cells showed plastic adhesion capacity and were able to support differentiation and growth of various hematopoietic cell lines [24].

The MSCs are multipotent human stromal/stem cells able to self-renew [25]. The general properties of MSCs include high proliferative potential and adhesion capacity, symmetric and asymmetric division, fibroblast-like morphology, easily induced differentiation, and the formation of colonies in a culture [25]. MSCs are able to differentiate into chondrocytes, fibroblasts, osteoblasts, adipocytes, and myoblasts [25]. The therapeutic effect of MSCs is based on their ability to secrete a number of signaling molecules, which simulate the functional activity of various targets in of the body [25]. The MSCs promote growth of hematopoietic progenitors by forming the specific microenvironment (niche) [26]. To date, the following markers have been detected on the surface of MSC cells isolated from various tissues: CD105, CD106, CD13, CD140b, CD140α, CD146, CD147, CD151, CD166, CD276, CD29, CD44, CD47, CD49, CD49E, CD54, CD56, CD59, CD73, CD81, CD9, CD90, CD90.1, CD98, HLA-I, Klf-4, NANOG, nestin, NG2, Oct-3, OCT-4, PDGF-R β, prolyl-4-hydroxylase, Sox-17, SSEA-3, STRO-1, and α-SMA [27].

Besides MSCs, MSC-derived exosomes can mediate cell activity and paracrine actions through carriage of proteins, lipids miRNAs, and mRNAs into target cells [28, 29]. Moreover, exogenous exosomes regulate expression of protein or target gene resulting regulation of function of the recipient cell [30]. It has been reported that exosomes have ability to stimulate effects of stem cell-like pro-regenerative in damaged regions directly [31].

The bone marrow is one of the main sources of MSCs, and although its aspiration is the most traumatic way among the MSC isolation procedures, it is the most evaluated approach for cell therapy [32]. The MSC number, differentiation potential, and the viability of the bone marrow MSCs (BM-MSCs) decrease with age [33]. In this regard, the ongoing search for alternative sources of MSC is going on. MSCs derived from the adipose tissue (AT-MSCs) can be alternative solution for BM-MSCs due to their comparable differentiation and therapeutic potential [34]. The adipose tissue is not only a metabolic reservoir for storage and formation of high-energy substrates, but also participates in hormone metabolism [35]. More profound study of the adipose tissue structure was performed by Martin Rodbell (1925–1998) who used techniques of proteolytic cleavage, mechanical grinding, and differential centrifugation for isolating 2 fractions of the adipose tissue—mature adipocytes and, more compact, cellular substance, which he later called stromal-vascular fraction (SSF) [36]. The SSF is heterogeneous and includes MSCs, preadipocytes, endothelial cells, pericytes, T cells, and M2 macrophages, fibroblasts, and pre-adipocytes [37]. In 2001, Zuk et al. [38] noted that properties of so-called AT-MSCs are similar to BM-MSCs. In an adult bone marrow, the ratio of MSCs to total cells is 1:10,000–100,000 [39], whereas in the adipose tissue, the ratio of MSCs to total cells is 1:30 [40]. AT-MSCs are easier and safer to obtain than BM-MSCs. The primary acquisition of AT-MSC is based on the manually procedure performed with the involvement of lipoaspirate fermentation technique [41]. The adipose tissue suitable for MSC isolation can be obtained either by skin flaps [42] or liposuction (LS) [43].

## AT-MSC collection approaches for treatment of NOA

LS as a surgical intervention is preferable for aspirating adipose tissue suitable for isolation of MSC [44]. Considering complications and the little traumatic impact of LS, no long-term postoperative rehabilitation of patients is required following this operation [45]. Currently, there are various techniques for LS implementation as new state-of-the-art equipment continues to emerge such as ultrasound or laser [46, 47]. The most popular option though is classical tumescent LS, where fat tissue in the donor area of the patient’s body is infiltrated with a mixture of sterile saline with low concentrations of local anesthetic and epinephrine [48]. The LS technique may have negative or positive effects on viability and quantity of MSC isolated from fat tissue [49, 50]. With classical LS, the negative pressure in the aspirator is reversely proportional to the number of isolated stem cells [51]. According to Matsumoto et al. [52], in the case of applying this type of surgical intervention, stem cells should be processed no later than 1 day after the extraction of the fat material from the body, since storage of the fatty substrate at a room temperature decreases number of viable stem cells. Small portions of autologous adipose tissue extracted from the patient’s body with a syringe are easily processed for MSC isolation, whereas the processing of large volume of aspirated fat is associated with certain difficulties [53].

With classical LS, the aspirate is separated into 3 layers: top fatty layer contains homogenized mature adipocytes destroyed during the operation; the middle layer is intact adipose tissue and the bottom layer contains residuals of the solution infiltrated into the patient’s tissue before surgery with plasma and blood cells [54]. Both top and bottom layers are removed from the container before processing aspirated fat [54]. The remaining middle layer is washed in sterile phosphate buffer containing antibacterial and antimycotic agents to avoid microbial contamination of the material [54]. Next, the adipose tissue is lysed in sterile collagenase solution to release the components of the SSF containing stem cells [55]. Different types of enzyme are used, but collagenase type IA is the most effective for MSC isolation [56]. Currently, considering the side effects of enzymatic approaches on the MSCs, non-enzymatic explant at isolation methods has been developed [57].

Despite large numbers of registered preclinical and clinical studies, safety of MSC-related therapies has remained the major concern for clinicians. The main risks of MSCs are proinflammatory properties, tumorigenicity, and fibrosis [58]. Among them, tumorigenicity is the most serious and many studies have shown that MSCs have the ability to converse into tumors as well as the ability to trigger tumor development [59]. The excessive productions of cytokines by MSC, such as growth factors and chemokines, directly act on surface receptors of cancer cells, thereby regulating tumor enhancement [60].

## MSC therapy of azoospermia from bench to bed

MSC transplantation is a relatively new therapy proposed to induce spermatogenesis and treat male infertility [61]. Since MSC are involved in processes such as cell survival, proliferation, migration, angiogenesis, and immune modulation, these cells are considered as an ideal material for azoospermia treatment. Achieving this therapeutic method for treatment of NOA using MSC needs evaluation of in vitro and in vivo studies as well as possibility of clinical trials with this purpose.

## In vitro studies on MSC and spermatogenesis

Some studies have indicated that embryonic stem cells very similar to MSCs found in the testes [62]. These cells are located in the basal layer of the testicular seminiferous tubules, and they can divide asymmetrically and give rise to progenitor cells. These cells survive chemotherapy and can trigger germinative cell differentiation [63]. They, therefore, serve as a reserve storage for stem cell population [64]. It is likely that the interaction between these cells and the transplanted MSC plays a crucial role in the fertility restoration.

A certain combination of growth factors, chemical components, genetic manipulations, and/or co-culture with other cells can be used to induce the differentiation of MSCs into the male (Table 1) or female germ cell epithelium (Table 2). For differentiation of various types of MSCs into male germ cells, different types of differentiation induction method have been developed as follows: (1) retinoic acid, (2) growth factors, (3) minerals, (4) co-culture, (5) conditioned media, (6) magnetic field, and (7) gene over-expression (Table 1). The results of in vitro studies have been published demonstrating that NOA can be restored through MSC transplantation. Furthermore, differentiation of AT-MSCs into male germ cells suggests that cell therapy can help reverse pathological changes in the testicular seminiferous tubules.

**Table 1:** Differentiation of mesenchymal stromal/stem cells (MSC) into male germ cells in vitro

MSC source	Source age	Species	Inducer	References
Adipose tissue	Adult	Dog	BMP4	[65]
Adipose tissue	Adult	Dog	CD61 overexpression	[66]
Adipose tissue	Adult	Goat	BOULE overexpression DAZL overexpression STRA8 overexpression	[67]
Adipose tissue	Adult	Human	Retinoic acid	[68]
Adipose tissue	Adult	Mouse	BMP4 EGF GDNF LIF Retinoic acid	[69]
Adipose tissue	Adult	Mouse	Sertoli cells co-culture Retinoic acid Testosterone	[70]
Adipose tissue	Adult	Mouse	Testicular cell conditioned medium Retinoic acid	[71]
Amniotic membrane	Fetal	Human	Retinoic acid	[72]
Amniotic membrane	Fetal	Mouse	BMP4 Retinoic acid	[73]
Bone marrow	Adult	Goat	BMP4 Retinoic acid	[74]
Bone marrow	Adult	Human	Retinoic acid Sertoli cell-conditioned medium	[75]
Bone marrow	Adult	Human	Retinoic acid	[76]
Bone marrow	Adult	Mouse	BMP4	[77, 78]
Bone marrow Adipose tissue	Adult	Mouse	BMP4 Retinoic acid	[79]
Bone marrow	Adult	Mouse	BMP4 Retinoic acid	[80]
Bone marrow	Adult	Mouse	Retinoic acid	[81–83]
Bone marrow	Adult	Mouse	Sertoli cell-condition medium	[84]
Bone marrow	Adult	Mouse	Static magnetic field BMP4	[85]
Bone marrow	Adult	Mouse	Retinoic acid Testicular cell co-culture	[86]
Bone marrow	Adult	Rat	bFGF LIF Retinoic acid	[87]
Bone marrow	Adult	Rat	Retinoic acid	[88]
Bone marrow	Adult	Rat	Sertoli cell co-culture	[89]
Bone marrow	Adult	Sheep	Inorganic zinc (sulfate)	[90]
Bone marrow	Adult	Sheep	Inorganic zinc (sulfate) Organic zinc (acetate) Retinoic acid	[91]
Bone marrow	Adult	Sheep	Retinoic acid TGF-β1	[92]
Bone marrow	Adult	Sheep	Retinoic acid	[93]
Bone marrow	Adult	Sheep	TGFb1 BMP4 BMP8b	[94]
Bone marrow	Fetal	Human	Retinoic acid Testicular extracts	[95]
Lung	Fetal	Human	Retinoic acid	[96]
Umbilical cord	Fetal	Human	BMP4 Retinoic acid	[97, 98]
Umbilical cord	Fetal	Human	BMP4	[99]
Umbilical cord	Fetal	Human	pCD61-CAGG-TRIP-pur (oCD61) plasmid	[100]
Umbilical cord	Fetal	Human	Testicular cell co-culture	[101]
Wharton’s jelly	Fetal	Human	BMP4 Testicular cell-conditioned medium Placental cell-conditioned medium Retinoic acid	[102]
Wharton’s jelly	Fetal	Human	BMP4 Placenta cell co-culture Retinoic acid	[103]
Wharton’s jelly	Fetal	Human	Retinoic acid Testosterone Testicular cell-conditioned medium	[104]
Wharton’s jelly	Fetal	Human	Retinoic acid	[105, 106]
Wharton’s jelly	Fetal	Human	Sertoli cell co-culture	[107]

*Abbreviations*: *bFGF* basic fibroblast growth factor, *BMP* bone morphogenetic protein, *EGF* epidermal growth factor, *GDNF* glial cell line-derived neurotrophic factor, *LIF* leukemia inhibitory factor, *TGFb1* transforming growth factor-beta 1

**Table 2:** Differentiation of mesenchymal stromal/stem cells (MSC) into female germ cells in vitro

MSC source	Source age	Species	Inducer	References
Adipose tissue Ovary Skin	Adult	Pig	Follicular fluid	[108]
Amniotic membrane Chorion Umbilical cord	Fetal	Human	BMP4	[109]
Follicular fluid	Adult	Human	BMP15	[110]
Menstrual blood	Adult	Human	Polylactic acid Multi-wall carbon nanotubes	[111]
Menstrual blood	Adult	Human	Follicular fluid	[112]
Muscle	Fetal	Pig	Follicular fluid	[113]
Ovary	Adult	Mouse	Oct4 overexpression	[114]
Ovary	Fetal	Cow	BMP4 BMP2 Follicular fluid	[115]
Peritoneum	Adult	Mouse	Human follicular fluid Human cumulus-conditioned medium	[116]
Skin	Adult	Pig	Follicular fluid	[117]
Umbilical cord	Fetal	Goat	Follicular fluid	[118]
Umbilical cord	Fetal	Human	Follicular fluid	[119]
Wharton’s jelly	Fetal	Human	Follicular fluid Cumulus cell-conditioned medium	[120]

*Abbreviations*: *B MP* bone morphogenetic protein

## MSC therapy in animal model of azoospermia

MSCs transplanted into the testes of chemical or surgical NOA animal models showed both induction of spermatogenesis and/or differentiation of MSCs into germ cells (Table 3). MSC transplantation improved the expression of germ cell markers in the testes and can be proposed as a suitable method for the treatment of infertility. Several possible mechanisms of testicular function restoration during MSC-induced tissue regeneration have been shown: (1) MSCs may be involved in the suppression of antisperm antibodies (ASA) [147]; (2) MSCs can reduce factors that lead to infertility through reduction of apoptosis [127]; (3) MSCs can reduce oxidative stress [139]; (4) MSCs can stimulate testosterone production [126] with differentiation into Laydig cells [148]; (5) MSCs can differentiate into target cells [133]; (6) the transplanted cells secrete growth factors such as bone morphogenetic proteins (BMPs) and transforming growth factor beta (TGF-β), which are male germ cell inducing factors with ability to stimulate restoration of the recipient’s cellular function [149]; (7) MSCs connect with endogenous cells, restoring the function of damaged cells [150]; (8) MSCs reverse the glycolysis and glycogenesis imbalance in sperm by regulating Akt/glycogen synthase kinase 3 (GSK3) axis [151]; and (9) MSCs can alter expression of some spermatogenesis-related miRNAs and their target genes [134].

**Table 3:** Azoospermia treated with mesenchymal stromal/stem cells in in vivo model studies

Source	Transplantation	Donor species	Therapeutics	Recipient species	Modeling	References
Adipose tissue	Allotransplant	Hamster	Cell	Hamster	Busulfan	[121]
Adipose tissue	Allotransplant	Mouse	Cell Exosome	Mouse	Busulfan	[122]
Adipose tissue	Allotransplant	Rat	Cell	Rat	Busulfan	[70, 123, 124]
Adipose tissue	Allotransplant	Rat	Cell	Rat	Cisplatin	[125]
Adipose tissue	Xenotransplant	Human	Cell	Rat	Torsion	[126]
Amnion	Allotransplant	Mouse	Cell	Mouse	Busulfan	[127]
Bone marrow	Allotransplant	Guinea pig	Cell	Guinea pig	Busulfan	[128]
Bone marrow	Allotransplant	Hamster	Cell	Hamster	Busulfan	[129]
Bone marrow	Allotransplant	Mouse	Cell	Mouse	Busulfan	[130, 131]
Bone marrow	Allotransplant	Mouse	Cell Exosome	Mouse	Busulfan	[122]
Bone marrow	Allotransplant	Mouse	Cell	Mouse	Cisplatin	[132]
Bone marrow	Allotransplant	Rat	Cell	Rat	Busulfan	[87, 89, 133–138]
Bone marrow	Allotransplant	Rat	Cell	Rat	Doxorubicin	[139]
Bone marrow	Allotransplant	Rat	Cell	Rat	Lead nitrate	[140]
Bone marrow	Allotransplant	Rat	Cell	Rat	Torsion	[141]
Bone marrow	Xenotransplant	Goat	Cell	Mouse	Busulfan	[142]
Umbilical cord	Xenotransplant	Human	Cell	Mouse	Busulfan	[143–145]
Urine	Allotransplant	Mouse	Cell Exosome	Mouse	Busulfan	[146]

## MSC therapy of azoospermia patients

Studies on in vitro differentiation of MSCs to germ cells and MSC therapy of animal models of azoospermia have showed the possibility of using MSC therapy to treat azoospermia in humans. Various clinical trials for the treatment of infertility in reproductive diseases in both women and men have been recorded or completed (Table 4). However, no studies have been published to treat azoospermia with the help of MSCs except an abstract from Jordan scientists demonstrating therapeutic effects of intratesticular injections of CD34/CD133 BM-MSCs in azoospermia men. At the same time, based on the information available in the US National Library of Medicine and in the Iranian Registry of Clinical Trials, 6 studies (Table 4) and 1 study (IRCT20190519043634N1), respectively, have been recruited for this purpose.

**Table 4:** Clinical trials on mesenchymal stromal/stem cells-based therapy for female and male reproductive diseases (U. S. National Library of Medicine)

Sex	Disease/syndrome	Phase	Date	Country	Source	Transplantation	Stage	CT code
Female	Atrophic endometrium	2	2019	Russia	Bone marrow	Autotransplant	Completed	NCT03166189
Female	Fistula vagina	1	2020	United States	ND	Autotransplant	Completed	NCT03220243
Female	Intrauterine adhesions Endometrial dysplasia	4	2014	China	Bone marrow	Autotransplant	ND	NCT02204358
Female	Intrauterine adhesions	ND	2014	China	Umbilical cord	Allotransplant	Completed	NCT02313415
Female	Intraventricular hemorrhage	2	2017	Korea	Umbilical cord	Allotransplant	Recruiting	NCT02890953
Female	Ovarian cancer	1	2019	United States	ND	Autotransplant	Completed	NCT02530047
Female	Ovarian disease	1&2	2015	Jordan	Bone marrow	Autotransplant	Active	NCT03069209
Female	Premature ovarian failure	1&2	2018	China	Umbilical cord	Allotransplant	Completed	NCT02644447
Female	Premature ovarian failure	1&2	2014	Egypt	Bone marrow	Autotransplant	ND	NCT02696889
Female	Premature ovarian failure	ND	2016	United States	Bone marrow	Autotransplant	Active	NCT02696889
Female	Thin endometrium	1	2018	China	Umbilical cord	Allotransplant	Recruiting	NCT03592849
Female	Thin endometrium	1	2020	Indonesia	Endometrium	Autotransplant	Recruiting	NCT04676269
Female	Uterine scar	2	2020	China	Umbilical cord	Allotransplant	Recruiting	NCT02968459
Female	Uterine scar	1	2020	China	Umbilical cord	Allotransplant	Recruiting	NCT03181087
Female	Uterus injury	2	2020	China	Umbilical cord	Allotransplant	Recruiting	NCT03386708
Male	Azoospermia	ND	2015	Egypt	Bone marrow	Autotransplant	Completed	NCT02414295
Male	Azoospermia	1&2	2014	Egypt	Bone marrow	Autotransplant	ND	NCT02025270
Male	Azoospermia	ND	2013	Egypt	Bone marrow	Autotransplant	Recruiting	NCT02008799
Male	Azoospermia	1&2	2014	Egypt	Bone marrow	Autotransplant	Recruiting	NCT02041910
Male	Azoospermia	1&2	2015	Jordan	Bone marrow	Autotransplant	Recruiting	NCT02641769
Male	Azoospermia Oligospermia	2	2018	Russia	Adipose tissue	Autotransplant	Recruiting	NCT03762967
Male	Erectile dysfunction	1	2018	Jordan	Wharton’s Jelly	Allotransplant	Completed	NCT02945449
Male	Erectile dysfunction	1&2	2019	Jordan	Wharton’s Jelly	Allotransplant	Completed	NCT03751735
Male	Erectile dysfunction	1	2018	Korea	Bone marrow	Autotransplant	Completed	NCT02344849
Male	Erectile dysfunction	2	2020	Korea	Bone marrow	Autotransplant	Recruiting	NCT04594850

*ND* no data

## Conclusions

The potential of MSCs in restoration of fertility in patients with NOA has been shown in this systematic review. Mastering and successfully applying this technique in clinical practice can help a vast group of patients to revive spermatogenesis and enjoy fatherhood. Based on the current knowledge answering to this important question “which MSCs source have a better therapeutic potential to azoospermia?” is not easy. Lack of comparing studies between the MSCs’ sources for treatment of azoospermia in the three layers of in vitro and in vivo studies and clinical trials made it difficult to rank the cell sources. By the way, considering the efficiency of cell isolation and complications of achieving a good cell source including higher number of cell yield, lower surgical manipulations, and similarity of donor cells and recipient, we can suggest adipose tissue-derived MSCs for treatment of azoospermia. However, other MSC sources may also be efficient for cell therapy of azoospermia.

## Data Availability

Not applicable.

## Abbreviations

ASA: Antisperm antibodies
AT-MSC: Adipose tissue-derived mesenchymal stromal/stem cell
BM-MSC: Bone marrow-derived mesenchymal stromal/stem cell
BMP: Bone morphogenetic protein
FSH: Follicle-stimulating hormone
GSK3: Glycogen synthase kinase 3
ICSI: Intracytoplasmic sperm injection
IVF: In vitro fertilization
LH: Luteinizing hormone
LS: Liposuction
MSC: Mesenchymal stromal/stem cell
NOA: Non-obstructive azoospermia
SHBG: Sex hormone-binding globulin
SSF: Stromal-vascular fraction
TESE: Testicular sperm extraction
TGF-β: Transforming growth factor beta
WHO: World Health Organization
